# Inconclusive role of human papillomavirus infection in breast cancer

**DOI:** 10.1186/s13027-015-0029-6

**Published:** 2015-10-26

**Authors:** Yi Zhou, Jinyuan Li, Yuting Ji, Ming Ren, Bo Pang, Ming Chu, Lanlan Wei

**Affiliations:** Department of Thyroid and Breast Diseases, The Central Laboratory in The First Affiliated Hospital of Harbin Medical University, Harbin, 150001 Heilongjiang China; Department of Microbiology, Harbin Medical University, Immunity and Infection, Pathogenic Biology Key Laboratory in Heilongjiang Province, Harbin, 150081 Heilongjiang China; Department of Neurosurgery, The First Affiliated Hospital, Harbin Medical University, Harbin, 150001 Heilongjiang China

**Keywords:** Human papillomavirus, Breast cancer, Epidemiology, Meta-analysis

## Abstract

**Background:**

Epidemiological studies have examined the association between human papillomavirus (HPV) and breast cancer, but the findings are inconclusive. This study aimed to detect the prevalence of HPV in breast cancer tissue in patients from northeastern China and define the association between HPV and breast cancer using meta-analysis.

**Methods:**

Polymerase chain reaction (PCR) was used to test cutaneous or mucosal HPV DNA sequence in 77 breast cancer samples and 77 corresponding adjacent normal tissues. The prevalence of HPV in breast cancer was estimated by pooling data from 38 studies. A meta-analysis of 16 case–control studies was conducted to investigate the association between HPV and breast cancer.

**Results:**

We did not find HPV DNA sequence in any of the 154 tissue specimens we tested. However, our meta-analysis revealed a HPV prevalence of 30.30 % (95 % confidence interval [CI] = 22.30–38.40 %) among breast cancer cases; most of these involved high-risk HPV types (35.50 %, 95 % *CI* = 25.00–46.10 %). HPV prevalence in breast cancer varied by geographic region, publication period, and PCR detection method. An increased risk of breast cancer was observed in association with exposure to HPV (odds ratio [OR] = 3.24, 95 % *CI* = 1.59–6.57), which was influenced by geographic region, HPV DNA source, PCR primer used, and publication period.

**Conclusions:**

HPV, especially high-risk HPV types, may be associated with an increased risk of breast cancer, and this association varies dramatically among geographic regions.

## Background

Breast cancer is the leading cause of cancer death in females worldwide, and its incidence and mortality rates have been rising in many Asian countries [1]. Although genetic susceptibility represents a risk factor for breast cancer, the etiologic factors that determine breast cancer risk have not been completely defined. Recently, increasing evidence has indicated that some viruses, especially human papillomavirus (HPV), may be involved in the pathogenesis of breast cancer [2–4].

It is well known that infection with specific types of HPV can cause cervical cancer. Additional evidence indicates a role for these high-risk HPV types in anal cancer, in up to 50 % of other anogenital cancers, and in 25–30 % of head and neck cancers [5]. The association of HPV with breast cancer was indicated when Band et al. reported that HPV could immortalize normal human mammary epithelial cells and reduce their growth factor requirements [6]. Later, HPV-16 DNA was detected in 29.4 % of breast carcinomas by polymerase chain reaction (PCR) [7]. Up to now the presence of HPV DNA has been repeatedly detected in 0 to 86 % of breast cancer [2–4, 8–40]; this large range indicates an inconsistent association of HPV with breast cancer. Considering that multiple primers are used to detect HPV DNA in breast cancers, we hypothesized that this inconsistency may partially result from the heterogeneity of primers used in HPV detection. Although both type-specific primers and general primers have been used in previous studies, most of these were designed for mucosal HPV types [10, 11, 13, 16, 20, 21]. Thus, cutaneous HPV types might escape detection.

The aim of our study was to detect any cutaneous or mucosal HPV types in breast cancer using two set of primers: MY09/11 [41], which targets mucosal HPV types, and FAP 59/64 [42], which targets cutaneous HPV types. Given the controversy among reports regarding the relationship between HPV and breast cancer, we systematically reviewed published studies of HPV and breast cancer to investigate the global prevalence and type distribution of HPV in breast cancer, to determine potential factors related to HPV prevalence, and to examine the evidence regarding the association of HPV infection and breast cancer risk.

## Results

### Molecular study

**Fig. 1:**
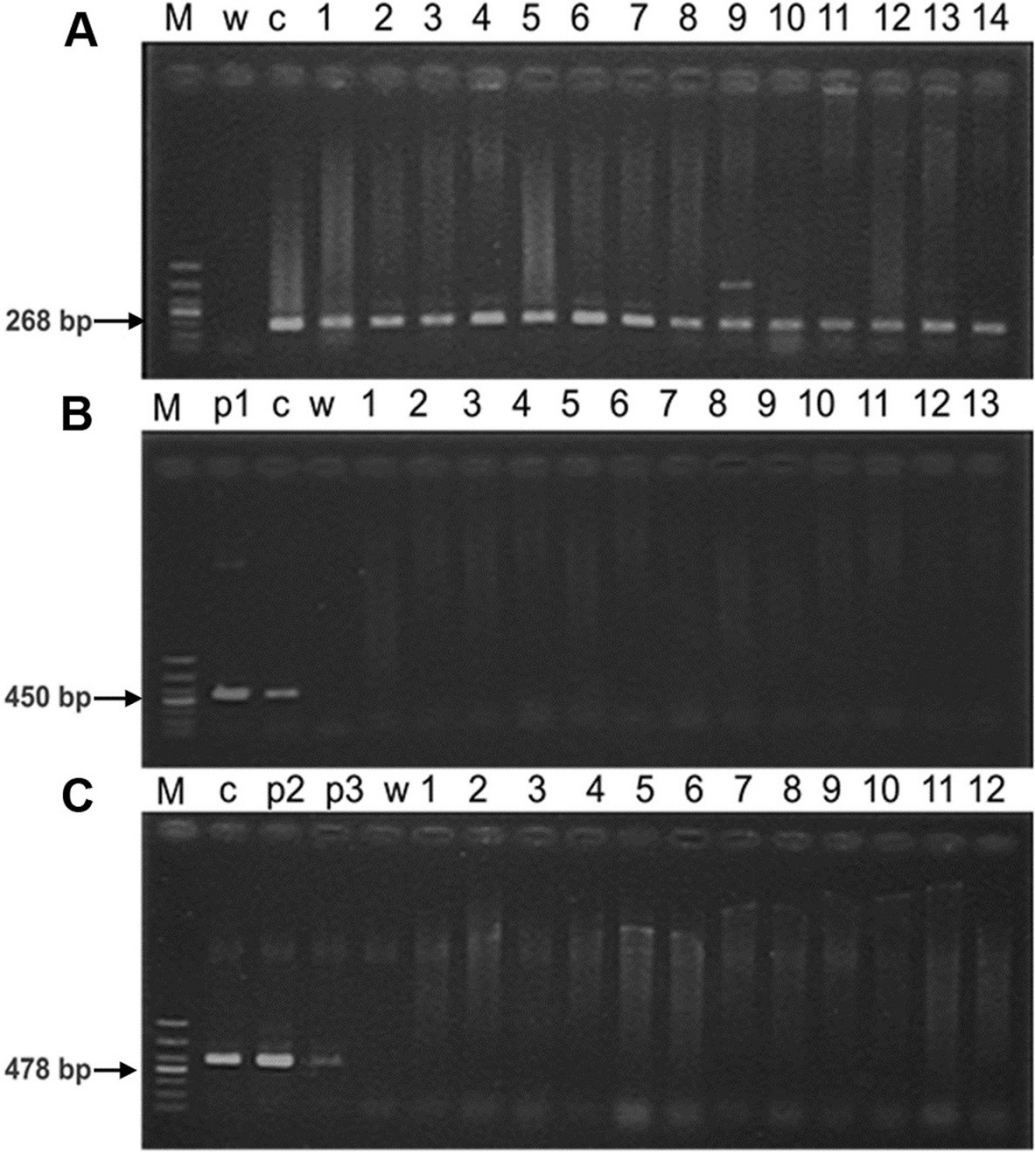
Identification the quality of genomic DNA from breast cancer biopsy and detection of HPV from them by PCR. **a** PCR amplification of a 268-bp fragment of human β-globin gene. **b** PCR amplification of a 450-bp fragment in the HPV L1 region detected using MY09/11 primers. **c** PCR amplification of a 478-bp fragment in the HPV L1 region detected using FAP59/64 primers. Lane M: DL 1000 DNA marker (TaKaRa). Lane W: Sterile water (negtive control). Lane c: DNA from HPV-16 positive cervical tissues. Lane p1: pBS322/HPV16 plasmid; Lane p2: pBS322/HPV8 plasmid; Lane p3: pBS322/HPV11 plasmid (positive control). Lanes 1–14: Breast cancer biopsy DNA

Of the 77 patients (age range: 39–74 years; mean: 49 years; *SE* = 9.43) selected for our molecular study, 61 had been diagnosed with invasive ductal carcinomas. The clinical characteristics of the breast cancer samples were shown in Table 1. None of the patients reported a family history of any other cancer. DNA isolated from all of these cases underwent detection of HPV infection by PCR. All samples were positive for β-globin gene amplification (Fig. 1a), indicating that DNA was available for molecular analysis. However, no HPV DNA was detected using either mucosal consensus primers (MY09/11) or cutaneous consensus primers (FAP59/64) in any of the 77 breast tumor tissues or 77 normal adjacent tissues (Fig. 1b and c). Weak bands between 400 and 500 bp appeared in two breast cancer samples, but the results of three extra PCR runs of the first-cycle products or DNA from these specimens were negative (data not shown).

**Table 1:** Clinical characteristics of breast cancer tissues

Patient no.	Age	Tumor type	Tumor grade	No. of LN mets	% ER staining	% PR staining
1	49	ILC	III	18	1	0
2	55	IDC	II	1	NA	NA
4	43	DCIS	I	0	90	70
5	44	IDC,ILC	II	0	90	1
6	59	IDC	NA	3	90	60
7	63	IDC	II	0	90	70
8	42	DCIS	II	3	90	90
9	54	IDC	II	9	95	95
10	62	IDC	III	0	0	0
11	61	IDC	III	0	95	5
12	39	IDC	II	14	80	95
13	60	IDC	III	0	1	1
14	40	IDC	III	13	0	3
15	57	IDC	II	0	90	75
16	58	IDC	II	1	95	80
17	54	IDC	II	0	NA	NA
18	39	IDC	II	0	90	60
19	37	IDC	II	5	90	80
20	36	IDC	III	0	40	80
21	49	IDC	III	5	80	40
22	44	IDC	III	0	NA	NA
23	39	IDC	III	4	30	50
24	52	IDC	III	0	20	80
25	46	IDC	II	0	0	80
26	48	IDC	II	0	90	0
27	37	IDC	III	28	0	1
28	48	IDC	II	10	NA	NA
29	46	IDC	II	0	0	0
30	38	IDC	III	0	0	50
31	51	DCIS	D2	0	0	80
32	52	IDC	III	0	80	20
33	35	DCIS	D3	0	90	70
34	38	DCIS	D3	4	0	50
35	45	IDC	III	0	0	1
36	64	IDC	III	NA	NA	NA
37	57	IDC	II	1	90	90
38	45	IDC	II	5	95	95
39	50	IDC	III	5	85	90
40	43	IDC	II	0		
41	47	DCIS	D3	0	NA	NA
42	52	DCIS	NA	0	85	70
43	49	IDC	III	4		
44	48	ILC	II	10	-	-
45	50	IDC	II	NA	NA	NA
46	68	IDC,MBC	II	5	90	50
47	63	IDC,MBC	III	0	85	85
48	53	IDC	III	21	85	5
49	31	IDC	III	0	0	1
50	68	IDC	III	0	0	0
51	53	IDC	III	0	5	3
52	69	IDC	III	0	1	1
53	54	IDC	II	10	90	50
54	40	IDC	II	0	80	0
55	51	IDC	II	0	80	50
56	43	IDC	II	0	65	30
57	56	IDC	II	0	70	85
58	37	IDC	II	3	30	90
59	31	DCIS	III	15	95	30
60	48	ILC	II	0	40	60
61	74	DCIS	III	4	90	15
62	53	IDC	II	0	NA	NA
636	40	IDC	III	0	100	5
4	45	IDC	III	0	50	90
65	32	IDC	III	1	1	1
66	56	IDC	II	8	1	0
67	55	ILC	III	7	0	0
68	55	ILC	II	6	80	90
69	42	IDC	II	0	10	90
70	57	IDC	II	0	0	90
71	44	IDC	II	1	90	90
72	46	IDC	II	0	0	0
73	38	IDC	II	0	70	10
74	53	ILC	II	NA	NA	NA
75	47	IDC	II	0	50	10
76	61	IDC	III	3	0	30
77	42	IDC	II	0	40	70

*LN mets* lymph node metastasis, *% ER staining* percentage of tumour cells stained for oestrogen receptor, *% PR staining* percentage of tumour cells stained for progesterone receptor, *IDC* invasive ductal carcinoma, *ILC* invasive lobular carcinoma, *DCIS* ductal carcinoma in situ, *MBC* mucinous breast adenocarcinoma, *NA* data not available

### The overall and type-specific prevalence of HPV in breast cancer and factors impacting the prevalence of HPV in breast cancer

**Fig. 2:**
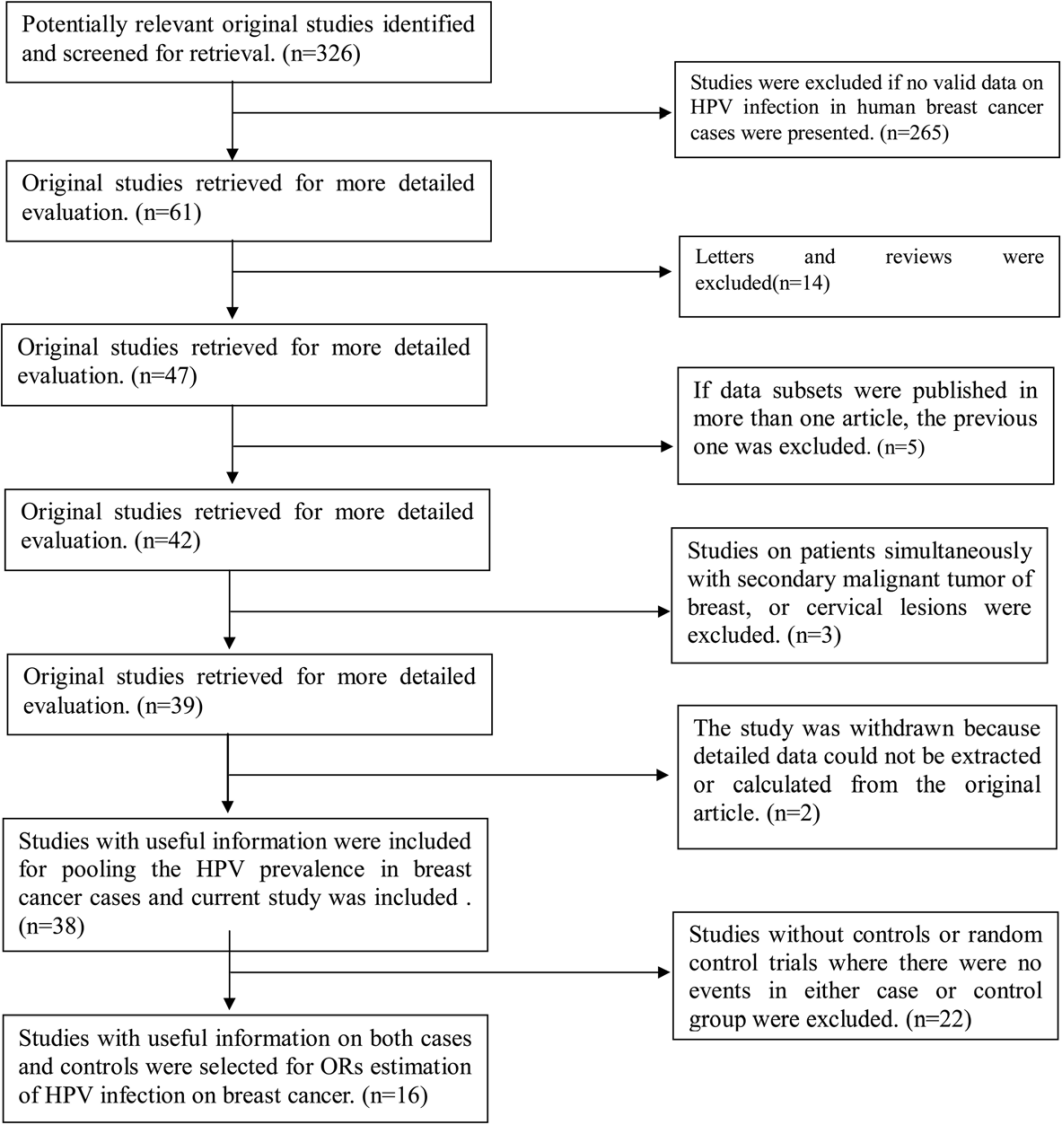
Flow chart of identifying the articles related to HPV and breast cancer

A total of 326 published records were retrieved using the key words mentioned previously (Fig. 2). Among those publications, 38 studies [2–4, 7–40] were pooled to calculate the HPV prevalence in breast cancer. The selected studies comprised a total of 2569 breast cancer cases, and the majority came from Asia (51.96 %) and South America (20.32 %). The number of cases investigated in each study varied from 17 to 228, and the prevalence of HPV ranged from 0 to 86 %.

The pooled prevalence of HPV in breast cancer across the 38 studies was 30.30 % (95 % *CI* = 22.30–38.40 %). Stratified by region, Oceania yielded the highest HPV prevalence in breast cancer, with 44.30 % (95 % *CI* = 33.50–55.00 %), followed by Asia with 35.70 % (95 % *CI* = 20.60–50.80 %), Europe with 32.60 % (95 % *CI* = 6.50–58.70 %), South America with 14.60 % (95 % *CI* = 5.80–23.50 %), and North America with 10.70 % (95 % *CI* = 4.40–17.00 %). When the unconditional logit regression model was introduced to compare HPV prevalence in breast cancer among different geographic regions, the result remained consistent: Oceanian breast cancer patients exhibited the highest HPV prevalence, and the priority of this prevalence was statistically significant (*P* < 0.001) (Table 2). HPV prevalence was found to be higher when HPV DNA was extracted from fresh tissues (37.30 %, 95 % *CI* = 20.10–54.50 %) than when HPV DNA was extracted from paraffin-embedded tissues (27.20 %, 95 % *CI* = 17.90–36.40 %), but this priority of prevalence was statistically insignificant (*p* = 0.08) (Table 2).

**Table 2:** HPV prevalence in breast cancer cases across region, HPV DNA source, and publication period

Variable	No. of studies	No. of cases	Proportion(%)	HPV prevalence (%) (95 % CI)	*P*	OR (95%CI)	Adjusted OR^a^ ( 95 % CI)
Total	38	2569	100	30.30(22.30–38.40)		…	…
Region					<0.001		
Oceania	3	130	5.06	44.30(33.50–55.00)		Ref.	Ref.
Asia	16	1335	51.96	35.70(20.60–50.80)		0.24(0.17–0.35)	0.21(0.14–0.32)
Europe	9	467	18.18	32.60(6.50–58.70)		0.17(0.11–0.27)	0.09(0.05–0.14)
South America	7	522	20.32	14.60(5.80–23.50)		0.14(0.09–0.21)	0.07(0.04–0.11)
North America	3	115	4.48	10.70(4.40–17.00)		0.14(0.07–0.28)	0.06(0.03–0.13)
HPV DNA source					0.08		
Fixed tissue	24	1537	59.83	27.20(17.90–36.40)		Ref.	Ref.
Fresh tissue	14	1032	40.17	37.30(20.10–54.50)		0.82(0.66–1.03)	0.46(0.36–0.60)
Publication period					0		
1992-2006	14	665	25.89	42.20(25.60–58.80)		Ref.	Ref.
2007-2012	24	1904	74.11	22.60(14.40–30.70)		0.37(0.30–0.46)	0.26(0.20–0.33)

*CI* confidence ratio, *OR* odds ratio, *Ref*. reference

^a^Adjusted for region, HPV DNA source and publication date

A cumulative meta-analysis was conducted to investigate the impact of publication period on HPV prevalence in breast cancer, and we found that the HPV detection rate between 2007 and 2012 (22.60 %, 95 % *CI* = 14.40–30.70 %) was dramatically lower than that between 1992 and 2006 (42.20 %, 95 % *CI* = 25.60–58.80 %) (*p* < 0.001) (Table 2).

PCR was the dominant method used to detect HPV in breast cancer; it was used in 31.30 % (95 % *CI* = 22.60–39.90 %) of the studies we reviewed, while HPV prevalence was 5.30 % (95 % *CI* = 2.40–8.30 %) using other methods, such as *in situ* hybridization and Southern blotting (Table 3). Multiple primers, including broad-spectrum PCR primers, type-specific PCR primers, and a combination of these, were used in PCR-based methods of detecting HPV. The HPV prevalence rate was 30.00 % (95 % *CI* = 18.30–41.70 %) when broad-spectrum PCR primers were used; it was 31.90 % (95 % *CI* = 13.00–50.80 %) when type-specific PCR primers were used; and it was 35.30 % (95 % *CI* = 11.50 –59.00 %) when a combination of these two types of primers was used. Because most studies dealt with invasive ductal cancer as well as non-invasive cancer, the detection rates of HPV in invasive ductal cancer were pooled, yielding HPV prevalence of 32.90 % (95 % *CI* = 21.50–44.20 %) compared with non-invasive cancer, which showed a prevalence rate of 22.00 % (95 % *CI* = 12.40–31.50 %) (Table 3).

**Table 3:** HPV prevalence in breast cancer cases by detection method and histological type

Variable	No. of studies	No. of cases	HPV prevalence (%) (95 % CI)
Detection method			
PCR-based method	37	2415	31.30(22.60–39.90)
Broad-spectrum primers	20	1102	30.00(18.30–41.70)
Type-specific primers	9	481	31.90(13.00–50.80)
Combined primers	8	832	35.30(11.50–59.00)
Non-PCR-based method^a^	6	466	5.30(2.40–8.30)
Histological type			
IDC	27	1536	32.90(21.50–44.20)
Other^b^	21	329	22.00(12.40–31.50)

*CI* confidence interval, *PCR* polymerase chain reaction, *IDC* invasive ductal carcinomas

^a^Others included *in situ* hybridization and Southern blotting

^b^Others included ductal carcinoma in situ, intraductal papilloma, medullary carcinoma, and mixed or unclear histological types

Although multiple HPV types were determined in breast cancer cases across the 38 studies in our meta-analysis, the five most common HPV types, in decreasing order of prevalence, were the following: HPV-16 (30.70 %; 95 % *CI* = 20.50–41.00 %), HPV-33 (25.60 %; 95 % *CI* = 7.10–44.00 %), HPV-11 (17.20 %; 95 % *CI* = 1.00–33.30 %), HPV-18 (9.90 %; 95 % *CI* = 6.20–13.70 %), and HPV-6 (6.30 %; 95 % *CI* = 2.00–10.50 %) (Table 4). Classified by HPV oncogenic features, the prevalence of high-risk HPV types was 35.50 % (95 % *CI* = 25.00–46.10 %), much higher than the prevalence of low-risk HPV types, which was 11.70 % (95 % *CI* = 5.80–17.70 %).

**Table 4:** Prevalence of Overall and Individual Human Papillomavirus (HPV) Types

HPV type	No. of studies	No. of cases	HPV prevalence (%) (95 % CI)
Total	38	2569	30.30(22.30–38.40)
High-risk^a^	18	1169	35.50(25.00–46.10)
Low-risk^b^	9	616	11.70(5.80–17.70)
Presence of individual type in breast cancer cases			
HPV-6	6	339	6.30(2.00–10.50)
HPV-11	4	134	17.20(1.00–33.30)
HPV-16	16	1029	30.70(20.50–41.00)
HPV-18	15	976	9.90(6.20–13.70)
HPV-31	3	334	3.00(0.00–6.10)
HPV-33	4	331	25.60(7.10–44.00)

^a^High-risk types included HPV-16, −18, −31, −33, −35, −51, −56, −58, −59, −66,-70,-73and −82

^**b**^Low-risk types included HPV-4,-6 ,-11,-24,-27,-57,and −87

### The association between HPV infection and breast cancer risk

**Fig. 3:**
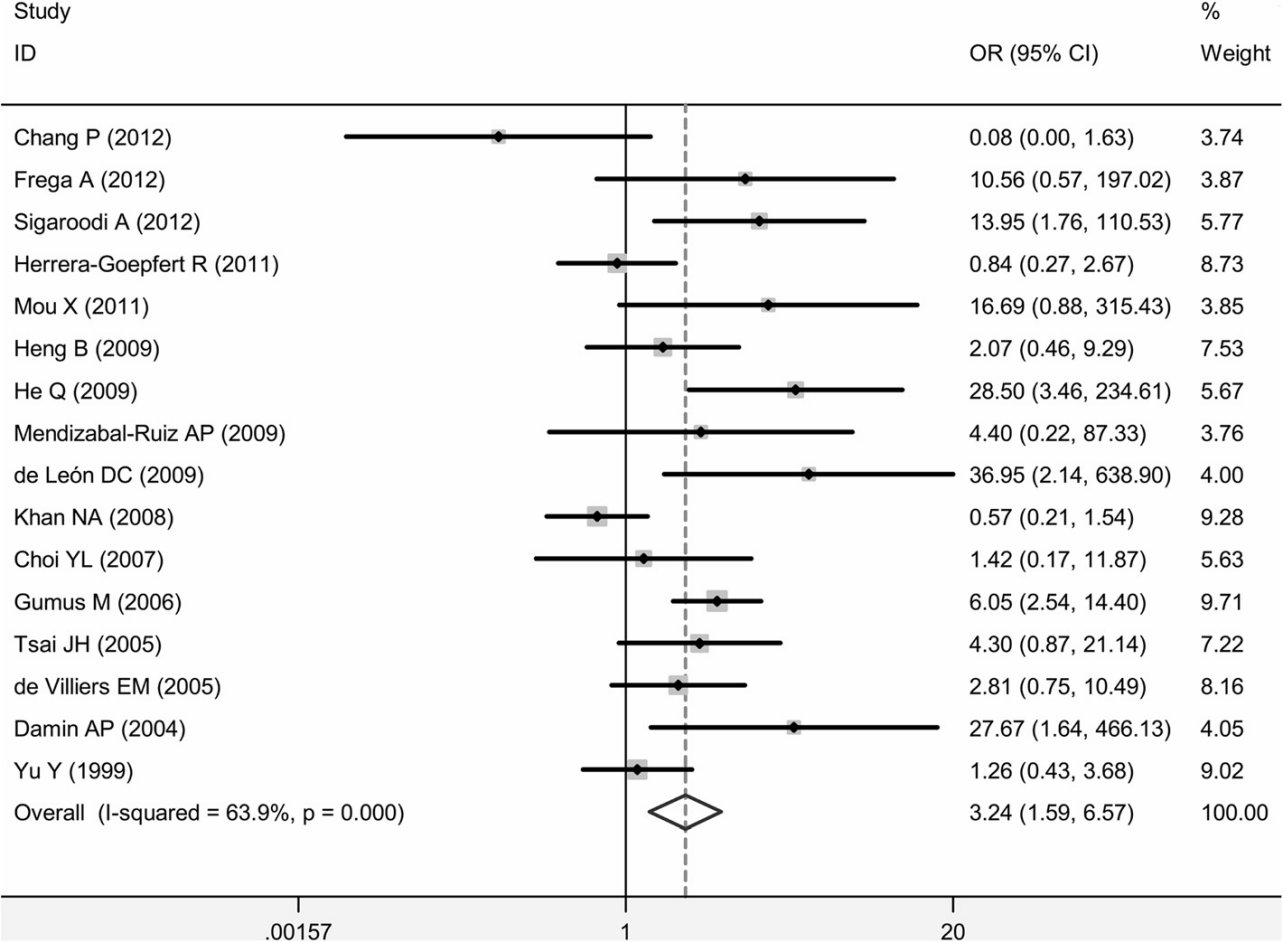
Forest plots of studies evaluating odds ratio of HPV infection to breast cancer risk

A total of 16 case–control studies were included in our evaluation of the association between HPV infection and breast cancer risk (Fig. 1). Due to existing heterogeneity (*p* = 0.000 for heterogeneity test, *I*^*2*^ = 63.9 %), a random model was chosen to calculate the combined OR and its 95 % CI across the 16 studies. When these 16 studies were pooled in the meta-analysis, results showed that HPV infection was associated with higher risk for breast cancer (OR =3.24, 95 % *CI* = 1.59–6.57, *p* < 0.001) (Fig. 3). Egger’s and Begg’s tests were performed to assess the publication bias; the results of both indicated no statistical significance (*p* = 0.16 and 0.45, respectively).

Considering that substantial heterogeneity was observed among the 16 studies for HPV infection and breast cancer, the prevalence of HPV infection in breast cancer was further evaluated by subgroup analysis. The analyzed subgroups were defined according to the main features of pooled studies: geographical region, DNA source, detection method (i.e., PCR primer), and publication period (Table 5). In the subgroup of geographical regions, between-study heterogeneity was absent (*p* = 0.611 for heterogeneity test, *I*^*2*^ = 0.0 %) in Oceania and Europe, but pooled OR remained the same (*OR* = 3.16, 95 % *CI* = 1.27–7.90, *p* < 0.001). When ORs were grouped by DNA source, however, significantly higher risk for breast cancer was revealed among fresh-tissue methods (*OR* = 7.88, 95% *CI* = 3.99–15.60, *p* < 0.001) without between-study heterogeneity. Similarly, breast cancer risk was greater with HPV infection in studies published between 1992 and 2006 (*OR* = 4.12, 95% *CI* = 2.42–7.02, *p* < 0.001) compared with studies published between 2007 and 2012. HPV infection detected using broad-spectrum PCR primers was associated with higher risk for breast cancer (*OR* = 5.67, 95 % *CI* = 3.40–9.45, *p* < 0.001), while HPV infection detected using combined PCR primers showed the opposite result (*OR* = 0.68, 95 % *CI* = 0.32–1.45, *p* < 0.001).

**Table 5:** Associations Between HPV prevalence and breast cancer Grouped by Selected Factors

Variable	No. of studies	Case vs Control		*P* for heterogeneity
		OR(95 % CI)^a^	OR(95%CI)^b^	
all	16	3.20(2.28–4.51)	3.24(1.59–6.57)	0.000
Region				
Asia	9	2.81(1.84–4.28)	2.87(1.05–7.85)	0.000
South America	4	4.70(2.18–10.14)	6.46(0.62–67.88)	0.007
Europe and Oceania	3	3.16(1.27–7.90)	…	0.611
DNA source				
Fixed tissue	12	2.31(1.55–3.45)	2.23(0.99–5.00)	0.004
Fresh tissue	4	7.88(3.99–15.60)	…	0.458
PCR primers				
Broad-spectrum primers	10	5.66(3.40–9.45)	…	0.566
Type-specific primers	4	3.34(1.72–6.47)	3.12(0.29–33.52)	0.002
Combined primers	2	0.68(0.32–1.45)	…	0.614
Publication period				
1992–2006	6	4.12(2.42–7.02)	…	0.111
2007–2012	10	2.69(1.73–4.20)	3.10(1.07–8.96)	0.000

*CI* confidence ratio, *OR* odds ratio

^a^Fixed effects model

^b^Random effects model

## Discussion

The role of HPV in breast cancer has been controversial; thus, a PCR-based strategy as well as a meta-analysis were conducted to investigate the association between HPV and breast cancer. In present study, 77 breast cancer samples and 77 adjacent normal tissues all tested negative for HPV. However, in our meta-analysis of 38 studies from 20 countries published since 1992, we found the overall HPV prevalence in women with breast cancer to be 30.30 % (95 % *CI* = 22.30–38.40 %). Furthermore, the prevalence estimate varied by geographic region, publication period, and PCR detection method. Our meta-analysis of 16 pooled case–control studies revealed that HPV infection is associated with breast cancer risk.

In our molecular study, both cutaneous and mucosal primers were applied to detect HPV DNA in breast tumors, but no specimen was positive for HPV. Other studies [9, 37] from China in which HPV DNA was detected in breast tumors have reported similar findings, suggesting that the HPV infection rate in breast cancer in Chinese patients is low and that HPV infection rate varies by geographic region. To support this idea, 38 studies [2–4, 7–40] from different regions were pooled to calculate the prevalence of HPV infection in breast cancer and to explore the factors impacting it.

The prevalence of HPV infection in breast cancer ranged from 10.70 % in North America to 44.30 % in Oceania. The geographic differences of HPV infection rates may be related to the distinctly different cervical cancer burdens across different continents [43]. In 1999, Hennig et al. reported that HPV-associated cervical neoplasia might be the original site of HPV infection from which the virus could be transported to the breast [44]. We found that HPV prevalence in frozen specimens was higher than that in paraffin-embedded tissues, but the difference was statistically insignificant (*P* = 0.08). Interestingly, the HPV detection rate of studies published between 1992 and 2006 was remarkably higher than that of studies published between 2007 and 2012. This can be explained by the fact that the latter generally included more breast cancer cases and that the specificity of detection advanced over time, thus yielding a conservative result.

In present study, HPV prevalence in 77 breast cancer samples and 77 adjacent normal tissues was zero, which is lower than that estimated by the meta-analysis included in this report. However, this is consistent with the results showed by stratified analysis, i.e. , HPV infection rate varies by geographic region and publication period (shown in Table 2). The geographic differences of HPV infection rates may be related to the distinctly different cervical cancer burdens across different continents, since cervix is proposed to be the original site of HPV infection from which HPV could be transported to the breast. In addition, due to the limitations of statistical software, 11 studies (references 8–12, 19–21, 27, 29, 30), as well as the present PCR-based study, showing no HPV prevalence in breast cancer, were not included in the pool used to estimate overall HPV prevalence. Thus, our pooled results showed a relatively strong combined HPV prevalence.

PCR emerged as the most effective method of detecting HPV in breast cancer, and combining general primers and specific primers revealed the highest HPV prevalence, due to the diversity of HPV types that co-occur with breast cancer. In our meta-analysis, HPV frequency in invasive carcinomas was higher than that in non-invasive carcinomas, but the difference was not statistically significant, suggesting that HPV infection may not be directly associated with the progression of breast cancer. Although multiple HPV types have been detected in breast cancer, high-risk HPV types are more frequently detected than low-risk HPV types. These results imply that the use of primers targeting high-risk HPV will increase the detection rate of HPV in breast cancer tissue.

Although the pooled estimate from 16 randomized controlled trials revealed that HPV infection resulted in an increased risk of breast cancer, the association was not strong enough to prove that HPV plays a causal role in breast cancer development. To identify the definitive role of HPV in breast cancer development, further studies are required to investigate the temporal association between the virus and breast cancer, the integration in the host genome, the expression of viral oncoproteins, the traits of HPV-positive breast cancer, and the route of breast infection.

Our meta-analysis has several limitations. First, due to the limitations of statistical software, 11 studies [8–12, 19–21, 27, 29, 30], as well as the present PCR-based study, showing HPV prevalence in breast cancer was zero, were not included in the pool used to estimate overall HPV prevalence; thus, our pooled results showed a relatively strong combined HPV prevalence. Interestingly, of 885 cases from the 11 studies that detected no HPV, 306 [9, 12, 20] cases (34.5 %) were from Asia and in them PCR was the only method used to detect HPV DNA. These results reflect those of the current PCR-based study. Second, substantial heterogeneity was observed when the pooled HPV prevalence was calculated across different continents. But unconditional logit regression revealed variables, including geographic region and publication period, that can only explain part of the existing heterogeneity. Third, some evidence supports the idea that some clinical features, including age and estrogen receptors, are linked to the HPV detection rate in breast cancer [33], and we cannot rule out the possibility of such an effect. Detailed information about these clinical characteristics were unavailable in the studies used in our meta-analysis.

Although two meta-analyses [45, 46] have been published regarding the correlation between HPV infection and the risk of breast cancer, our meta-analysis included a greater number of publications as well as more detailed information on HPV prevalence in breast cancer. Thus, our study has yielded more valid results.

## Conclusions

HPV infection, especially high-risk HPV types, may be associated with an increased risk of breast cancer, and the association varies dramatically among geographic regions.

## Methods

### Sample collection

A total of 77 breast cancer specimens and 77 corresponding paraneoplastic breast tissues were collected at the time of operation and were frozen immediately at −80 °C. These samples were obtained from patients diagnosed between February 2011 and May 2012 at the First Affiliated Hospital of Harbin Medical University (Harbin, China). All of the patients were women living in northeastern China, and none had a history of cervical carcinoma. Informed consent according to the criteria of Harbin Medical University was obtained from all patients, and this study was approved by the Institutional Research Board of Harbin Medical University (No. HMUIRB20120002).

### DNA extraction and PCR amplification

DNA was extracted from fresh frozen breast tissues as previously described, using SDS lysis/proteinase-K digestion, phenol/chloroform extraction, and ethanol precipitation. The DNA quality was confirmed by the amplification of a 268-bp fragment of human β-globin gene using and primers G073 (5′-GAAGAGCCAAG GACAGGTAC-3′) and G074 (5′-CAACTTCATCCACGTTCACC-3′). To avoid contamination between samples, the cutting blade was changed after the sectioning of each sample.

DNA samples were then screened for the presence of HPV by PCR using two sets of primers: MY09/11 and FAP59/64. Consensus primers MY09 (5′-CGTCCMARRGGAWACTGATC-3′) and MY11 (5′-GCMCAGGGWCATAAY AATGG-3′) amplify a 450-bp fragment in the highly conserved region in L1 of mucosal HPV types (annealing temperature 50 °C). Degenerated primers FAP59 (5′-TAACWGTIGGICAYCCWTATT-3′) and FAP64 (5′-CCWATATCWVHCATIT CICCATC-3′) can detect a 478-bp fragment in the L1 region of both mucosal and cutaneous HPV types (annealing temperature 55 °C). PCR was performed in a volume of 20 μL, containing 100 ng of extracted DNA, 1.67 mM MgCl_2_, 200 μM dNTPs, 0.4 μM of each primer and 0.5 U Taq DNA polymerase (TaKaRa, Japan), and reaction buffer (10 × PCR buffer, TaKaRa, Japan), according to the following conditions: 5 min at 94 °C, then 40 cycles of 5 s at 98 °C, 30s at 50 or 55 °C, and 60 s at 72 °C, followed by a 5-min extension at 72 °C in an automated thermocycler (Eppendorf, Germany). The full genomes of HPV-16, −11, and −8 were cloned in the pBS322 plasmid (donated by Michelle A. Ozbun), and HPV-18-positive cervical cancer specimens were used as positive controls; sterile water was used as a negative control. The PCR products were separated on 1.5 % agarose gel (Blowest, Spain) and visualized with ethidium bromide staining after electrophoresis.

### Search strategy and selection criteria for HPV and breast cancer

Eligible articles published from January 1989 to June 2013 were identified by an electronic PubMed search using the MeSH terms “human,” “papillomavirus,” and “breast carcinoma.” Additional articles from the reference lists of retrieved articles were also screened. The inclusion criteria of the studies were as follows: (1) detection of HPV in female breast cancer; (2) full text in English; (3) independent of other studies. The experimental results from the present molecular study were included in the meta-analysis.

### Data extraction for HPV and breast cancer

Two authors (Jinyuan Li and Lanlan Wei) independently extracted data and reached a consensus on all issues. Disagreements were discussed and resolved according to the inclusion criteria and consensus. The following data were extracted from each report: the first author, year of publication, country of origin, ethnicity, sample size (cases and controls), HPV prevalence, DNA source, detection method, PCR primers used, histological subtypes tested, and HPV types detected.

### Statistical analysis

An epidemiological review of the overall and type-specific HPV prevalence in breast cancer cases was conducted. Four high-risk HPV types were identified (HPV-16, −18, −31, −33), and two low-risk HPV types were identified (HPV-6 and −11). A meta-analysis was performed to explore the association between HPV infection and breast cancer risk in the form of a case (breast cancer tissues) versus control (normal breast cancer tissues, breast tumor adjacent tissues, or benign breast lesions) comparison. An unconditional logistic regression model was used to compare HPV prevalence according to the influential parameters: HPV DNA source, PCR primers used in detection, and publication period.

A fixed-effect model (Mantel-Haenszel method) and a random-effect model (DerSimonian and Laird method) were used to pool the case–control data. These two models provide similar results when between-study heterogeneity is absent; otherwise, the random-effect model is more appropriate. Between-study heterogeneity was tested using the χ^2^-based Q test, and heterogeneity was considered significant if *p* < 0.05. Subgroup analyses were further performed to explore the source of the existing heterogeneity. Publication bias was evaluated using the linear regression asymmetry tests designed by Egger et al. [47]. Analyses were carried out using Stata version 11 software (StataCorp, College Station, TX).
